# Optical Coherence Tomography Biomarkers of the Outer Blood—Retina Barrier in Patients with Diabetic Macular Oedema

**DOI:** 10.1155/2020/8880586

**Published:** 2020-10-13

**Authors:** Ioana Damian, Simona Delia Nicoara

**Affiliations:** ^1^Department of Ophthalmology, “Iuliu Hațieganu” University of Medicine and Pharmacy, 8 V. Babes str., 400012 Cluj-Napoca, Romania; ^2^Medical Doctoral School 1, Universitatii Str, 410087 Oradea, Romania; ^3^Clinic of Ophthalmology, Emergency County Hospital, 3 – 5 Clinicilor Str, 400006 Cluj-Napoca, Romania

## Abstract

**Background:**

Numerous studies confirmed the main role of the inner blood-retinal barrier in the development of Diabetic Macular Oedema (DMO). Lately, the focus of research shifted towards the external retinal barrier with potential involvement in the pathogenesis of DMO.

**Objective:**

We aim to identify the OCT changes of the external blood-retinal barrier in patients with DMO and to define them as biomarkers with predictive value. *Materials and method*. We set up retrospectively 3 groups of patients diagnosed with nonproliferative diabetic retinopathy (NPDR) and DMO, proliferative diabetic retinopathy (PDR) and DMO, and controls. We compared the RPE thickness in every quadrant between groups and performed correlations between best-corrected visual acuity (BCVA) and the thickness of the retinal layers. The Social Science Statistics platform was used for statistical tests.

**Results:**

The NPDR-DMO group consisted of 18 eyes, the PDR-DMO group consisted of 19 eyes, and the control group included 36 eyes. In the PDR-DMO group, RPE thickness was decreased in almost all quadrants (*p* < 0.001); in the NPDR-DMO group, only the central minimum and central maximum values of the RPE thickness were significantly different from the control group. We did not find any strong correlation between BCVA and the thickness of the retinal layers.

**Conclusion:**

The thickness of the RPE layer is an OCT biomarker able to predict the functioning of the outer BRB. Eyes with PDR-DMO exhibited decreased thickness of the RPE layer in almost all quadrants, highlighting the degenerative changes occurring in a hypoxic environment. The thickness of a specific layer could not be identified as a biomarker to correlate significantly with BCVA, most likely because we did not analyze specific morphologic features, such as continuity and reflectivity. The analysis of the RPE thickness could clarify the unexplained decrease of BCVA and predict early the evolution of DR.

## 1. Introduction

Diabetic macular oedema (DMO) is the main cause of visual impairment within the group of working-age population in developed countries [1]. DMO affects 1 in 15 patients diagnosed with diabetes mellitus (DM), and its prevalence is constantly increasing worldwide. Fluid accumulation in the macular area translates clinically by the decrease of visual acuity (VA), but also by difficulty with facial recognition, reading, or driving [1].

The retina is one of the most metabolically active tissues in the organism, requiring important amounts of glucose and lactose [2]. The need for two distinct blood-retinal barriers (BRB), inner and outer, confirms the complexity of the retina and enhances the need to maintain a homeostatic retinal microenvironment [2]. The primary role of the internal BRB's disruption in the pathogenesis of DMO was confirmed by numerous studies, but it is becoming more and more obvious that also outer BRB is involved in its evolution. Outer BRB separates the neural retina from the choroidal vascularisation which is responsible for approximately 80% of the ocular blood supply [2]. Retinal pigment epithelium (RPE) plays important roles in retinal metabolism: it provides nutrition for the photoreceptors, it removes the metabolic waste resulted from the phagocytosis of the photoreceptors' outer segments [3], and it is responsible for pumping the extravasated fluid from the internal retinal vessels towards the choriocapillaris, driven by the transport of Cl^−^ and K^+^ [4], thus filling the lack of lymphatics [5]. Furthermore, BRB is involved in the transport and recycle of docosahexaenoic acid, a major component of the photoreceptors [2]. The diabetic retina, characterised by a highly hypoxic environment, stimulates the overexpression of hypoxia-inducible factor (HIF-) 1*α* and of vascular endothelial growth factor (VEGF). VEGF is also responsible for the depletion of the occludin in RPE, with subsequent disruption of the tight junction's integrity in the outer BRB [6].

Electronic microscopy demonstrated the degeneration of RPE in DMO induced in animal models: shrank nuclei, reduced endoplasmic reticulum, in-folding of the cell membrane, altered melanosome, and even loss of RPE cells [5]. When electroretinogram was performed on a diabetic mice model, a decreased c wave was identified before the occurrence of photoreceptors' dysfunction [7]. Other studies that used fluorescein angiography-based technology distinguished endothelial barrier leakage from RPE barrier-specific leakage [2, 8]. In cell cultures, like RPE-51 and ARPE-19, VEGF upregulated ZO-1*α*^−^ and ZO-1*α*^+^ mRNA and proteins, causing an increased TER (transepithelial resistance) which is an indicator of RPE's barrier function. In addition, when soluble VEGF was neutralized with an antibody, it led to partial recovery of the RPE barrier's function [9]. Exposure of ARPE-19 cell line and primary human retinal pigment epithelial to hypoxia increased the secretion of IL-6 and IL-8 and also of VEGF, as shown by Arjama et al. [10], which describes the same environment as in a retina with proliferative diabetic retinopathy (PDR). When RPE proteome was analyzed in diabetic eyes without retinopathy, sixty-two percent of RPE's proteins involved in retinoid metabolism, regulating energy and chaperone proteins, were found to be altered. Moreover, they were also changed in nonretinal tissue, suggesting that RPE is compromised as part of the systemic impact of diabetes [11].

The aim of the present study was to investigate whether the thickness of RPE is modified in patients with DMO associated with nonproliferative diabetic retinopathy (NPDR) or proliferative diabetic retinopathy (PDR), using Spectral Domain-Optical Coherence Tomography (SD-OCT). The novelty of our approach comes from the observation that even if there is evidence in the literature that RPE thickness decreases in patients with DMO [12], there is no distinction so far between the cases with NPDR and PDR associated to the DMO.

The primary outcome of this research is to find out whether there was a difference between groups in RPE thickness. The secondary outcomes are to identify the differences between DMO with NPDR and DMO with PDR versus control. Furthermore, we aimed to investigate if there is a correlation between RPE and inner retinal thickness, photoreceptors, and central macular thickness (CMT). Finally, we intended to identify the OCT biomarkers that correlate best with BCVA: central macular thickness (CMT), inner retinal thickness, photoreceptor layer thickness, or RPE thickness.

## 2. Materials and Methods

### 2.1. Study Design

A retrospective, single-centre, observational, and comparative study was carried out. The study was approved by the Ethics Committee belonging to “Iuliu Hatieganu” University of Medicine and Pharmacy (IHUMP), Cluj-Napoca, Romania, and the study protocol adhered to the rules of the Declaration of Helsinki.

### 2.2. Study Sample

**Figure 1:**
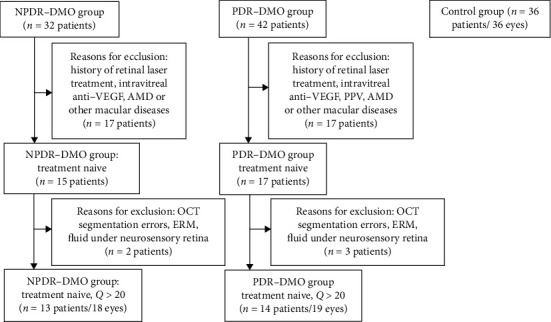
Flow diagram illustrating the study selection process. AMD: age-related macular degeneration; anti-VEGF: anti-vascular endothelial growth factor; PPV: pars plana vitrectomy; ERM: epiretinal membrane, Q: OCT segmentation quality; NPDR-DMO: nonproliferative diabetic retinopathy-diabetic macular oedema; PDR-DMO: proliferative diabetic retinopathy-diabetic macular oedema.

We included in the study the patients diagnosed with type 1 or type 2 DM and with NPDR or PDR associated with DMO. The patients were examined in the Department of Ophthalmology belonging to IHUMP, between January 2017 and September 2019. Patients with an ophthalmological examination in the same setting between July and September 2019, with no history of DM, were selected for the control group. Thus, we set up 3 groups of patients: NPDR with DMO, PDR with DMO, and control. The algorithm according to which the 3 groups were created is presented in Figure 1.

#### 2.2.1. Diabetic Retinopathy Group

An eye was eligible for diabetic retinopathy (DR) group if the following criteria were met: NPDR (level 20-53E of the Early Treatment Diabetic Retinopathy Study (ETDRS) classification) or PDR (61-65 of the ETDRS classification). All the recruitments were performed by an ophthalmologist with experience in medical retina, and they were validated by OCT (Spectralis HRA+OCT, Heidelberg Engineering, Heidelberg, Germany) examination. Patients with a history of vitreoretinal surgery, laser or anti-VEGF injections, AMD or other macular diseases, ocular trauma, lens or corneal opacification, vitreous hemorrhage, tractional retinal detachment, segmentation errors on OCT examination, OCT segmentation quality less than 20 db, or subretinal fluid were excluded. If a patient was confirmed with bilateral DR meeting the selection criteria, but different ETDRS stages, both eyes were included in the study.

#### 2.2.2. Control Group

Every eye included in the study had BCVA equal or above 20/40, refraction with spherical equivalent less than ±5 dpt and had undergone macular OCT imaging. Patients who were confirmed with DM, macular diseases, ocular trauma, glaucoma, significant opacification of the lens or cornea, segmentation errors on OCT examination, OCT segmentation quality less than 20 db were excluded. One eye was randomly selected for the final analysis for each patient within this group.

All participants underwent VA testing measured with the Snellen acuity chart, slit lamp biomicroscopy, dilated eye fundus examination.

The following baseline clinical characteristics were recorded: age, gender, BCVA, DR, and DMO classification.

Demographic data and ophthalmic examination were collected from the hospital's informatic system. OCT data were collected from the OCT database.

### 2.3. Assessment of OCT Data

OCT was performed using Spectralis OCT (Heidelberg Engineering, Inc., Heidelberg, Germany). The fast macular protocol was used: 25 raster lines per eye separated by 240 *μ*m, with a 20 × 20° scan and an automatic real mean value (ART value) set at 9. All scans were performed by the same experienced technician. Segmentation was automatically performed using the Spectralis software version 6.0. Only images with more than 20 db signal strength and with individual retinal layers that could be identified were used for the analysis. ETDRS macular maps were used to report macular thickness: 1, 3, and 6 mm concentric rings. The central 1 mm ring was defined as central thickness. The 3 mm ring, known as the intermediate, was divided into four quadrants: inner superior, inner inferior, inner nasal, and inner temporal, and the 6 mm ring, known as the outer ring, was divided into outer superior, outer inferior, outer nasal, and outer temporal. The numerical values such as thickness and volume recorded for each quadrant were used in the analysis.

**Figure 2:**
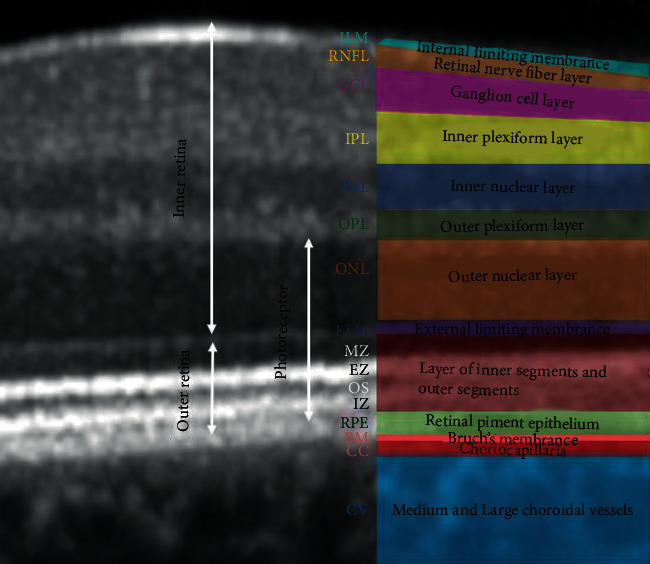
Retinal layer segmentation.

The boundaries between the retinal layers are illustrated in Figure 2. We define the following parameters: central macular thickness (CMT)—between ILM and Bruch's membrane; RPE layer—between the outer limit of photoreceptor layer (PR1/2) and Bruch's membrane; outer retina—between ELM and Bruch's membrane, and ONL (outer nuclear layer)—between the outer plexiform layer (OPL) and ELM. The inner retinal thickness was considered from ILM to ELM (Figure 2).

In order to check out the relationship between RPE and the photoreceptors, we approximated the thickness of the photoreceptor layer as follows: from the outer retina, we subtracted the RPE thickness to get the thickness of photoreceptors' inner and outer segments (PR 1/2); then, we added to PR 1/2 the thickness of ONL (rod and cone cell bodies). As a result, the boundaries of the photoreceptor layer are the inner limit of the RPE band and the outer limit of the OPL.

We further detailed the segmentation of the outer retina. Thus, we defined the inner segments of the photoreceptors (IS), the outer segments of the photoreceptors (OS), and the interdigitation zone (IZ). IS are divided into two parts: myoid zone (MZ) and ellipsoid zone (EZ). MZ is a hyporeflective region located between ELM and EZ. It corresponds to the myoid portion of the inner photoreceptors' segments. EZ is a hyperreflective band between MZ and OS, previously known as the junction between photoreceptors' inner and outer segments; it represents the ellipsoid layer of the outer portion of the inner photoreceptors' segments. The OS layer is a hyporeflective band between EZ and IZ. IZ is a hyperreflective band representing the contact between the apices of the RPE cells and the outer segments of the photoreceptors; it was previously called the cone outer segment tips (COST) and rod outer segment tips (ROST).

We defined the thickness of the inner quadrant as the average thickness from all the four inner sectors and the thickness of the external quadrant as the average thickness from all the four outer sectors.

The images were reviewed by the investigator before data analysis, and manual adjustments to retinal layer segmentation were made if necessary.

### 2.4. Statistical Analysis

In order to perform statistical analysis, the Snellen Visual Acuity fraction was converted into an approximate ETDRS letter score. Numerical variables are summarized with means and standard deviations, whereas the nominal variables are expressed in frequencies and percentages. The nonparametric Kruskal-Wallis test was applied to assess the differences between the thickness of retinal layers among groups followed by post hoc analysis with the Mann-Whitney test if an overall significance was found. We corrected for the effect of multiple comparisons by conducting a posteriori Bonferroni adjustment. The gender difference between the groups was compared using the chi-squared test.

The Spearman correlation coefficient was used for the detection of correlations between quantitative variables such as the different thickness of layers and VA or age.

*p* values ≤0.05 were considered statistically significant. The platform Social Science Statistics (https://www.socscistatistics.com/) was used to perform the tests.

## 3. Results

### 3.1. Demographics and Clinical Characteristics of the Study Samples

A total of 73 eyes were included in the analysis, as follows: 18 eyes within the NPDR-DMO group, 19 eyes within the PDR-DMO group, and 36 eyes within the control group. We used the Kruskal-Wallis test for age and BCVA and the chi-squared test for gender. No statistically significant difference emerged regarding the age and gender distribution between the groups. BCVA was significantly different between NPDR-DMO and control (*p* < 0.00001), PDR-DMO and control (*p* < 0.00001), but not significantly different between NPDR-DMO and PDR-DMO (*p* = 0.3125). The baseline characteristics of these patients are presented in Table 1.

### 3.2. RPE Thickness and Volume

The RPE thickness and volume in every quadrant (see Table 2) were compared between the groups, and significant results such as internal quadrant (*p* < 0.00001), central subfield (*p* = 0.026), central minimum (*p* < 0.00001), central maximum (*p* < 0.00001), inner nasal (*p* = 0.0005), inner superior (*p* = 0.0002), inner inferior (*p* = 0.0017), outer nasal (*p* = 0.02), and outer superior (*p* = 0.009) were further analysed with the Mann-Whitney test.

**Figure 3:**
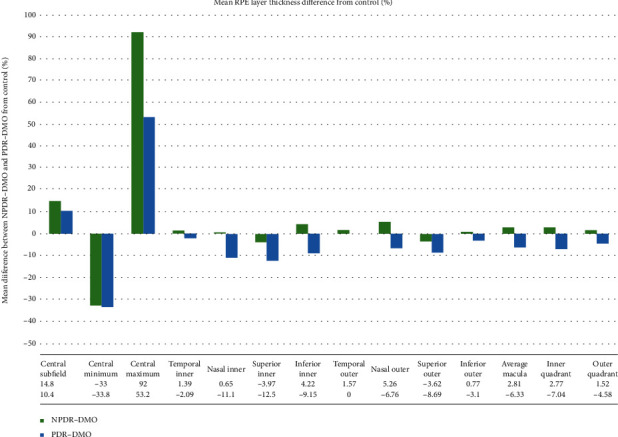
Mean RPE layer thickness difference (%) between the eyes from the control group and NPDR-DMO or PDR-DMO.

The mean RPE thickness in the eyes with PDR-DMO compared to controls was decreased in most quadrants: central minimum (-33.8%), temporal inner (-2.09%), nasal inner (-11.1%), superior inner (-12.5%), nasal outer (-6.76%), superior outer (-8.69%), inferior outer (-3.1%), average RPE (-6.33%), inner quadrant (-7.04%), and outer quadrant (-4.58%). In contrast, in the eyes with NPDR-DMO, the RPE thickness was decreased as compared to controls, for central minimum (-33%), superior inner (-3.97%), and superior outer (-3.62%), but increased for the remaining quadrants (see Figure 3).

After post hoc analysis with the Mann-Whitney test and Bonferroni adjustment, the differences between NPDR-DMO and controls were statistically significant for the central minimum (*p* < 0.00001) and central maximum (*p* < 0.00001) thickness values. Regarding PDR-DMO and controls, differences between central thickness (*p* = 0.00008), central minimum (*p* < 0.00001), central maximum (*p* < 0.00001), inner nasal quadrant (*p* = 0.00044), inner superior (*p* < 0.00001), inner inferior (*p* = 0.00058), and internal quadrant (*p* = 0.0014) were statistically significant. Between NPDR-DMO and PDR-DMO, only the thickness of the inner nasal quadrant (*p* = 0.009) was statistically different (see Table 3).

### 3.3. Correlations

**Figure 4:**
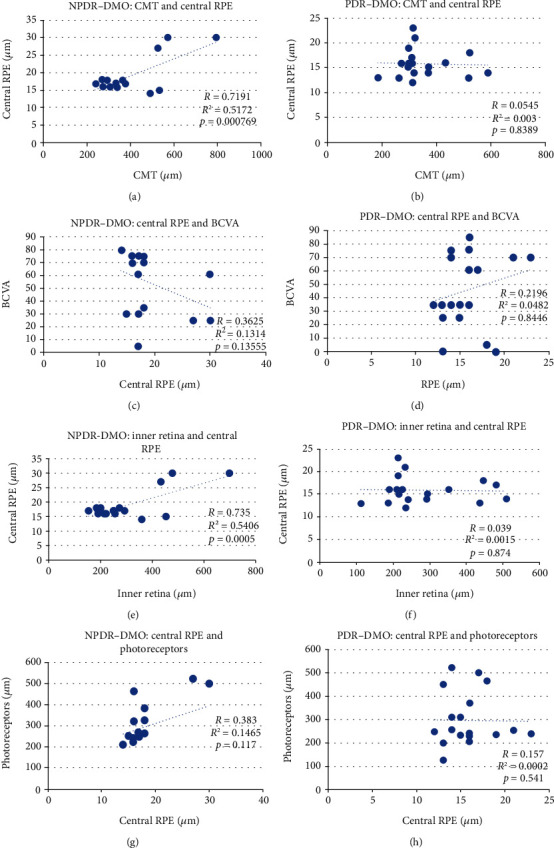
Scatterplots between different variables: (a) CMT and central RPE in NPDR-DMO; (b) CMT and central RPE in PDR-DMO; (c) central RPE and BCVA in NPDR-DMO; (d) central RPE and BCVA in PDR-DMO; (e) inner retina and central RPE in NPDR-DMO; (f) inner retina and central RPE in PDR-DMO; (g) central RPE and photoreceptors' thickness in NPDR-DMO; (h) central RPE and photoreceptors' thickness in PDR-DMO.

In the NPDR-DMO group, we identified a high positive correlation between CMT and central RPE (*r* = 0.719) (see Figure 4(a)), inner retina and central RPE (*r* = 0.735) (see Figure 4(e)), a low positive correlation between photoreceptors and central RPE (*r* = 0.383) (see Figure 4(g)), and a low negative correlation between the central RPE and BCVA (-0.362) (see Figure 4(c)), CMT and BCVA (-3.68), and the inner retina and BCVA (*r* = −0.3686) (see Table 4). In the PDR-DMO group, we found a low positive correlation between the outer retina and BCVA (*r* = 0.451). The remaining correlations were negligible (see Table 4). We compared photoreceptor thickness between the groups: NPDR-DMO vs. control: *p* < 0.00001; PDR-DMO vs. control: *p* < 0.00001; and NPDR-DMO vs. PDR-DMO: *p* = 0.4009.

## 4. Discussion

Since 1995, when the first study regarding the status of OCT in the diagnosis of macular diseases was published, this new technology has provided important insights into the pathophysiology and treatment of retinal diseases [13]. OCT has enhanced the ophthalmologist's understanding of retinal microstructure, to the extent that currently we are able to analyse the anatomy of the photoreceptors and RPE and to anticipate their functioning [14].

For a long time, CMT has been the only biomarker according to which macular oedema was analyzed. However, progress in OCT technology revealed other structural changes, like intraretinal cysts, the disintegration of the retinal structure, flattening of the central fovea, haemorrhages, hard exudates, and subretinal fluid [13].

Since age was similar within our groups, the differences in the thickness between layers cannot be assigned to an age-related diffuse loss of neural tissue, nor to an accumulation of excessive metabolic strain causing an increased thickness [15] or an optical “pseudothickening” due to hyperreflectivity [15].

In the context of increased retinal thickness, especially on the account of INL and OPL [16], the external layers such as RPE and photoreceptors seem to decrease, proving the complex pathogenetic mechanism of DMO.

In the PDR-DMO group, apart from CMT, the RPE thickness was decreased in all quadrants. The reason for this finding seems to be a disruption of the RPE-photoreceptors complex [12], possibly due to ischemia, as demonstrated by Reznicek et al. [17] and by Boynton et al. [12]: the thickness of the outer retinal layers, meaning RPE and photoreceptors, was slightly reduced by ±9 *μ*m and ± 8 *μ*m, respectively. Constant oxidative stress which is a feature of DR impairs autophagy (the removal of damaged organelles and protein aggregates from the same cell) and heterophagy (phagocytosis of exogenous photoreceptor outer segments in RPE cells), as proved by Kaarniranta et al. [18]. In contrast, in the NPDR-DMO group, the number of quadrants with decreased RPE thickness was lower as compared to the PDR-DMO group. This is a reasonable finding when considering that the level of inflammation and ischemia varies according to the stage of DR.

However, higher than the normal values were found occasionally when measuring RPE thickness, as proved within the groups of CMT in PDR-DMO and NPDR-DMO. One possible explanation is that over the RPE cells, new cells grow in order to compensate and to minimise the fluid leakage within the retina [5]. Another hypothesis is that the disturbance of the RPE cells' phagocytosis induces the accumulation of shed outer segments that are not timely engulfed in the RPE-photoreceptors' complex [19].

When we examined the RPE volume, in the PDR-DMO group in all quadrants, the values were decreased as compared to controls, but the differences were not statistically significant. In the NPDR-DMO group, in some quadrants, the volume was increased, whereas in other quadrants, it was decreased. This is probably due to the oedema within the layers and the lower ischemic status.

Besides its leading role in the diagnosis and monitoring of the response to treatment, OCT delivers biomarkers able to predict BCVA. Over time, multiple hypotheses were tested. The most frequently used OCT biomarker was CMT, but scenarios in which the normalization of CMT was not paralleled by the improvement of BCVA or with a modest correlation between the two variables were described [20]. Further on, the correlation between BCVA and the inner retina was evaluated; Sun et al. described the disorganization of the inner retinal layers and he named it DRIL. He proved that although associated with worse BCVA, it predicts better the BCVA outcome [21]. Later on, the integrity of ELM and IS/OS was found to be positively correlated with BCVA [22–26].

Taking into account the multiple roles played by the RPE for the normal functioning of the photoreceptors, the search for a correlation with BCVA is mandatory. In the PDR-DMO group, we found only a low positive correlation between the outer retina and BCVA. In the NPDR-DMO group, a low negative correlation was identified between CMT, central RPE thickness, inner retina thickness, and BCVA. Our results are limited by the analysis of cell thickness, not morphology. Therefore, thickness within the normal range is compatible with altered cellular anatomy. IS/OS and ELM are useful hallmarks to evaluate the integrity of the foveal photoreceptor layer, being closely associated with the final BCVA [27]. BCVA before treatment and photoreceptor status can predict the potential restoration of photoreceptor integrity and subsequent visual recovery in DMO [28].

Further on, we intended to find out if there is any correlation between the CMT and the central thickness of the RPE, namely, whether the RPE thickness will influence the CMT. In the NPDR-DMO group, the correlation was highly positive, whereas in the PDR-DMO group, it was negative, but negligible. This finding could be explained by a higher level of oedema within the retinal layers in the NPDR-DMO group, as compared to the PDR-DMO one.

We also set out to identify if there was any correlation between the internal and external retinal barriers, by approximating an overlap with the OCT layers: inner retina = internal BRB and RPE = external BRB. As Das et al. [21] have found, DRIL was strongly associated with the disruption of ELM and EZ, and the retinal thickness at the fovea (RTF) was increased in the presence of DRIL, suggesting that the inner retinal disorganization could be responsible for the disruption of the outer retinal architecture. They concluded that the breakdown of BRB in DMO could set the stage for the damage of ELM and EZ. We found a highly positive correlation between the thickness of the inner retina and the thickness of the central RPE in the NPDR-DMO group, but a low negative one in the PDR-DMO group. Therefore, it appeared obvious that in patients with DMO, the level of retinopathy is of utmost importance. Thereby, in NPDR, oedema involves the entire retina, whereas in PDR, macular oedema is driven mainly by ischemia and to a lesser extent by a vasogenic mechanism. Moreover, as Zhang et al. [29] underlined, high glucose promotes the production of reactive oxygen species (ROS) and cell apoptosis, and it inhibits mitophagy, whereas low glucose, although it induces ROS production and cell mitophagy, has a lesser impact on cell apoptosis and proliferation.

It is well known that RPE is a monolayer of pigmented cells that are vital for the photoreceptors' functioning, survival, and maintenance. After having proved the role of RPE damage in the pathogenesis of DMO, we aimed to quantify its effect on the photoreceptor layer. RPE and photoreceptor layers are regarded as a functional unit due to their interdependence. Structural and functional changes of this complex were found also in patients with DR without DMO [19]. When analysing the total thickness of the photoreceptors (inner and outer segment plus ONL), the decreased values we found in the PDR-DMO group and in the NPDR-DMO group could be attributed to a thinner PROS (photoreceptor outer segment) length in the context of a relative outer retinal hypoperfusion induced by hypoxia, as shown by Verma et al. [30]. As Nesper et al. [31] and Muir et al. [32] pointed out, the decrease of choroidal blood flow creates a hypoxic environment for the RPE and photoreceptor cells with subsequent disruption of phagocytosis and increased fragility of the RPE cells. In a feedback loop, more superoxide and soluble inflammatory factors are produced that aggravate the condition [19].

Ferreira et al. [33] have reported a thicker RPE layer and a thinner photoreceptor layer in patients with DM without DR, as opposed to the nondiabetic controls.

When comparing the results between studies, we must pay attention to the type of OCT machine and to the segmentation algorithm of the outer retina because different results could emerge [19]. Xia et al. reported that the increase of the RPE-photoreceptor thickness precedes the alterations of the retinal nerve fibre layer (RNFL) or of the ganglion cell layer (GCL) [19].

Our study has several limitations: the small sample size, the quantitative assessment of the RPE layer, and the selection bias. The strength of our study comes from the different approach of making the distinction between the NPDR and PDR within the group of patients with DMO. Our results add to previous research serving as evidence for the key part played by the changes in the RPE layer during the evolution of DR.

## 5. Conclusions

In the PDR-DMO group, apart from CMT, RPE thickness was significantly decreased in almost all quadrants in our series. In the NPDR-DMO group, the number of quadrants with significantly decreased RPE thickness was lower as compared to the PDR-DMO group, proving the key impact of DR staging on DMO.

In the PDR-DMO group, we found only a low positive correlation between the outer retina and BCVA. In the NPDR-DMO group, a low negative correlation was identified between the central RPE thickness and BCVA.

In the NPDR-DMO group, the correlation between the CMT and the central RPE thickness was highly positive, whereas in the PDR-DMO group, it was negative, but negligible.

The photoreceptors' thickness was significantly lower in both groups, PDR-DMO and NPDR-DMO.

Further and more refined studies are needed to provide definite OCT biomarkers by analyzing the outer BRB in patients with DMO.

## Figures and Tables

**Table 1:** Baseline characteristics of the patients included in the study.

	Control (*n* = 36)	NPDR-DMO (*n* = 18)	PDR-DMO (*n* = 19)	*p* value
Age, years	53.3 ± 14.19 CI 95% (11.51 to 18.51)	61 ± 8.57 CI 95% (6.43 to 12.85)	57.8 ± 9.56 CI 95% (7.23 to 14.14)	0.151
Gender (F/M)	20 (55.6%)/16 (44.4%)	9 (50%)/9 (50%)	13 (68.4%)/6 (31.6%)	0.497
BCVA, letters	83.75 ± 2.50 CI 95% (2.03 to 3.26)	55.52 ± 24.46 CI 95% (18.22 to 37.23)	45.94 ± 27.57 CI 95% (20.83 to 40.77)	<0.00001

^∗^The results are expressed as mean ± *SD* or frequency with percentages in parentheses. *N*: number; F: female; M: male; BCVA: best-corrected visual acuity; NPDR-DMO: nonproliferative diabetic retinopathy-diabetic macular oedema; PDR-DMO: proliferative diabetic retinopathy-diabetic macular oedema.

**Table 2:** RPE thickness and volume in each ETDRS macular map quadrant.

RPE	Control	NPDR-DMO	PDR-DMO	Kruskal-Wallis *p*
Central subfield (*μ*m)	16.2 ± 1.7	18.6 ± 6.9	15.8 ± 2.8	*0.026*
Central minimum (*μ*m)	12.4 ± 1.3	8.3 ± 4.3	8.2 ± 2.8	*<0.00001*
Central maximum (*μ*m)	21.4 ± 2.6	41.1 ± 27.3	32.8 ± 11.6	*<0.00001*
Central volume (mm^3^)	0.0106 ± 0.0023	0.0117 ± 0.0038	0.0111 ± 0.0031	0.972
Temporal inner quadrant (*μ*m)	14.3 ± 1.3	14.5 ± 1.7	14 ± 2.58	0.173
Temporal inner volume (mm^3^)	0.0218 ± 0.0038	0.0217 ± 0.0038	0.0216 ± 0.0050	0.993
Nasal inner quadrant (*μ*m)	15.3 ± 1.6	15.4 ± 1.5	13.6 ± 1.2	*0.0005*
Nasal inner volume (mm^3^)	0.0231 ± 0.0047	0.0233 ± 0.0048	0.0210 ± 0.0032	0.890
Superior inner quadrant (*μ*m)	15.1 ± 1.6	14.5 ± 2	13.2 ± 1.2	*0.0002*
Superior inner volume (mm^3^)	0.0222 ± 0.0042	0.0222 ± 0.0043	0.0205 ± 0.0023	0.928
Inferior inner quadrant (*μ*m)	14.2 ± 1.4	14.8 ± 3.8	12.9 ± 1	*0.0017*
Inferior inner volume (mm^3^)	0.0214 ± 0.0035	0.0217 ± 0.0051	0.02	0.956
Temporal outer quadrant (*μ*m)	12.7 ± 0.9	12.9 ± 0.8	12.7 ± 2.2	0.277
Temporal outer volume (mm^3^)	0.0669 ± 0.0052	0.0683 ± 0.0062	0.0658 ± 0.0126	0.798
Nasal outer quadrant (*μ*m)	13.3 ± 1.2	14 ± 4.1	12.4 ± 1.1	*0.020*
Nasal outer volume (mm^3^)	0.0075 ± 0.0711	0.075 ± 0.0218	0.0642 ± 0.0067	0.504
Superior outer quadrant (*μ*m)	13.8 ± 1.4	13.3 ± 1.2	12.6 ± 1.1	*0.009*
Superior outer volume (mm^3^)	0.0728 ± 0.0085	0.0706 ± 0.0072	0.0668 ± 0.0075	0.708
Inferior outer quadrant (*μ*m)	12.9 ± 1.1	13 ± 1.6	12.5 ± 1.1	0.120
Inferior outer volume (mm^3^)	0.0686 ± 0.006	0.0689 ± 0.0096	0.0674 ± 0.0148	0.832
Average thickness (*μ*m)	14.2 ± 1.1	14.6 ± 1.4	13.3 ± 0.9	0.316
Total volume (mm^3^)	0.3845 ± 0.0296	0.3917 ± 0.0371	0.3637 ± 0.0295	0.758
Internal quadrant (*μ*m)	14.4 ± 1.3	14.8 ± 1.4	13.4 ± 1.1	*<0.00001*
External quadrant (*μ*m)	13.1 ± 0.9	13.3 ± 1.4	12.5 ± 1.2	0.165

The results are expressed as mean ± *SD*. The italicized values indicate a statistically significant difference between the groups: *p* < 0.05. RPE: retinal pigment epithelium; NPDR-DMO: nonproliferative diabetic retinopathy-diabetic macular oedema; PDR-DMO: proliferative diabetic retinopathy-diabetic macular oedema.

**Table 3:** Post hoc analysis for ETDRS quadrants with a statistically significant difference after the Kruskal-Wallis test.

RPE thickness	Control vs. NPDR-DMO	Control vs. PDR-DMO	NPDR-DMO vs. PDR-DMO
Central subfield	0.039	*0.00008*	0.017
Central minimum	*<0.00001*	*<0.00001*	0.447
Central maximum	*<0.00001*	*<0.00001*	0.741
Nasal inner quadrant	0.936	*0.00044*	*0.0009*
Superior inner quadrant	0.322	*<0.00001*	0.022
Inferior inner quadrant	0.660	*0.00058*	0.009
Nasal outer quadrant	0.841	0.0110	0.019
Superior outer quadrant	0.208	0.0028	0.101
Internal quadrant	0.976	*0.0014*	0.003

The italicized values indicate a statistically significant difference between groups. *p* < 0.001 adjusted Bonferroni. RPE: retinal pigment epithelium; NPDR-DMO: nonproliferative diabetic retinopathy-diabetic macular oedema; PDR-DMO: proliferative diabetic retinopathy-diabetic macular oedema.

**Table 4:** Correlations between BCVA and retinal layers thickness.

Correlation	NPDR-DMO	*R* ^2^	*p*	PDR-DMO	*R* ^2^	*p*
Central RPE and BCVA	-0.362	0.131	0.153	0.220	0.048	0.845
Outer retina and BCVA	0.086	0.007	0.743	*0.451*	0.203	0.053
CMT and BCVA	-0.368	0.136	0.146	-0.119	0.014	0.654
Photoreceptors and BCVA	-0.0066	0	0.981	-0.102	0.010	0.687
Inner retina and BCVA	*-0.386*	0.149	0.127	-0.069	0.047	0.782
CMT and central RPE	*0.719*	0.517	0.0007	-0.054	0.003	0.839
Inner retina and central RPE	*0.735*	0.541	0.0002	-0.039	0.001	0.874
Photoreceptors and RPE	0.383	0.146	0.117	0.061	0.005	0.785

RPE: retinal pigment epithelium; NPDR-DMO: nonproliferative diabetic retinopathy-diabetic macular oedema; PDR-DMO: proliferative diabetic retinopathy-diabetic macular oedema; CMT: central macular thickness; BCVA: best-corrected visual acuity.

## Data Availability

The data used to support the findings of this study are available from the corresponding author upon request.
